# Caregiver Health Literacy and Healthcare Utilization Behaviors: A Prospective Study Comparing Caregivers of Orthopaedic Patients With Chronic Versus Acute Conditions

**DOI:** 10.1016/j.jposna.2026.100389

**Published:** 2026-05-06

**Authors:** Nora A. Galoustian, Michaela Juels, Alex Rueda, Aura Elias, Gabrielle Noullet, Anahit Malumyan, Lisa Su, Mauricio Silva, Rachel Mednick Thompson

**Affiliations:** 1 University of California Los Angeles David Geffen School of Medicine, Los Angeles, California, USA; 2 University of Southern California, Department of Epidemiology, Los Angeles, California, USA; 3 University of California, Department of Psychology, Los Angeles, California, USA; 4 Univeristy of California Los Angeles, Department of Public Health, Los Angeles, California, USA; 5 University of California Los Angeles Department of Orthopaedic Surgery, Los Angeles, California, USA; 6 Luskin Orthopaedic Institute for Children, Los Angeles, California, USA; 7 University of California San Diego Department of Orthopaedic Surgery, San Diego, California, USA; 8 Rady Children's Hospital Southern Family Center for Cerebral Palsy, San Diego, California, USA

**Keywords:** Health literacy, Pediatric orthopaedics, Cerebral palsy

## Abstract

**Background:**

Health literacy (HL) is the degree to which individuals comprehend information and services to inform health-related decisions and is a strong predictor of health outcomes. This study investigates whether increased exposure to the healthcare system equips caregivers of children with a chronic disease – cerebral palsy (CP) – with higher baseline HL compared to caregivers of otherwise healthy pediatric patients seeking acute surgical treatment.

**Methods:**

This prospective study utilized a single pediatric referral healthcare system over a 15-month period to recruit caregivers of pediatric patients with CP undergoing planned orthopaedic surgery (CP group) and caregivers of otherwise healthy pediatric patients undergoing planned surgical treatment for acute fractures (fracture group). Baseline HL was assessed pre-operatively with the Newest Vital Sign survey; charts were reviewed for no-shows, cancellations, and advice-only calls. Mann-Whitney U tests and pair-wise post hoc tests were utilized to compare cohorts.

**Results:**

Ninety patients were included for analysis, of which 37 (41.1%) were in the CP group and 53 (58.9%) were in the fracture group. There was no difference in baseline caregiver HL between cohorts (CP: 3.76 ± 1.62; fracture: 3.55 ± 2.09, *P* = .790). However, the fracture cohort had significantly higher no-show (CP: 0.03 ± 0.16; fracture: 0.49 ± 1.17, *P* = .008) and canceled appointments (CP: 1.16 ± 1.69; fracture: 1.94 ± 2.43, *P* = .032). Conversely, the CP group had significantly higher advice-only calls (CP: 2.51 ± 3.42; fracture: 0.06 ± 0.31, *P* < .001).

**Conclusion:**

Despite more interaction with the healthcare system, caregivers of children with CP do not have measurably higher HL compared to caregivers of children seeking acute, episodic fracture care. While these two cohorts demonstrate comparable baseline HL levels, their needs and utilization of healthcare differ considerably. These findings emphasize the importance of tailoring education to individual caregivers, providing counseling and resources at each visit, and ensuring caregiver comprehension regardless of patient status, system familiarity, or healthcare utilization.

**Key Concepts:**

(1) Caregivers of children with CP do not demonstrate higher baseline HL compared to caregivers of children with acute fractures.
(2) Despite similar HL scores, caregivers in the CP group made significantly more advice-only calls, suggesting greater post-operative care needs.
(3) Tailored educational support is essential, as healthcare system exposure alone does not equate to improved caregiver health literacy.

**Level of Evidence:**

II; Lesser-quality prospective study

## Introduction

Health literacy (HL) is the degree to which individuals have the ability to comprehend and access information and services to inform health-related decisions and actions for themselves and others. Poor HL is a predictor of adverse health outcomes including increased morbidity [1]. While HL has been explored in the context of chronic disease affecting adult populations, there are comparatively fewer studies exploring the implications of baseline health literacy on primary caregivers of children with special healthcare needs (CSHCN). This patient population may be at particular risk for inadequate exchange of health information, diminished understanding of treatment instructions, and poor adherence due to the complexity of their health care needs.

Children with cerebral palsy (CP) represent a subgroup within CSHCN with overlapping risk factors for vulnerability due to multiple medical comorbidities. Primary caregivers play a major role in health decision-making in this patient population. Caregivers of children with CP seek medical advice from primary care physicians and utilize urgent care centers more frequently than those without CP, both for CP-related issues and general pediatric medical needs [2]. While it is intuitive that ongoing exposure to the healthcare system would increase these caregivers’ understanding of the medical system, there is currently no evidence to support this assertion.

This study explores the relative effect of chronic healthcare system exposure on caregivers’ HL in this patient population. Our primary outcome is baseline health literacy among caregivers with differing levels of healthcare exposure. Our secondary outcome is healthcare utilization behaviors, comparing caregivers of children with cerebral palsy seeking surgical intervention to caregivers of otherwise healthy pediatric patients undergoing surgical treatment for acute fractures. We hypothesized that caregivers of children with chronic health needs would demonstrate higher baseline health literacy levels but engage in more healthcare utilization behaviors compared to caregivers of otherwise healthy pediatric patients.

## Materials and methods

### Study location and population

Between March 29, 2023 and July 1, 2024, caregivers of pediatric patients with CP undergoing orthopaedic surgery (CP group) and patients undergoing surgery for acute fractures (fracture group) were recruited from a single pediatric tertiary referral healthcare system. Inclusion criteria for the CP group were patients diagnosed with CP scheduled for planned orthopaedic surgery. For the fracture group, children without any underlying medical diagnoses scheduled for outpatient surgical fixation of acute extremity fractures were included. Patients over 18 years of age and those undergoing emergent surgical intervention were excluded. This study was approved by the University of California, Los Angeles Institutional Review Board (IRB #23-000108 and #22-000799); written informed consent was obtained from all caregivers prior to enrollment.

### Survey instrumentation

Caregivers were surveyed to determine baseline HL pre-operatively. HL was measured using the Newest Vital Sign (NVS), a validated six-item survey wherein a higher score corresponds with higher HL [3]. Patient Reported Outcomes Measurement Information System (PROMIS) Global Health scores were also collected. This survey is a validated seven-item tool assessing caregiver perceptions of overall patient health [4]. Scores above 50 indicate a perception of better health than the reference population. The survey was not used to compare absolute health between cohorts, but rather to reflect how caregiver perceptions of their child's health relate to their reported health literacy.

### Demographic and outcome variables

Demographic variables such as age, patient race, patient ethnicity, caregiver race, insurance status, caregiver primary language, and home address were collected via chart review. Healthcare utilization behaviors were reviewed and defined as the number of post-operative advice-only phone calls, appointment cancellations, and no-show appointments within ninety days post-operatively. Advice-only phone calls were only included if they were patient or caregiver-initiated and related to the patient's surgery or post-operative care. All advice-only calls were documented by the nurse who took these calls and captured via chart review in the Electronic Health Record. Home addresses were used to generate State Area Deprivation Indexes (ADI), a validated tool quantifying socioeconomic disadvantage. State ADI is divided into deciles (1-10), with higher scores indicating more disadvantage. More disadvantaged groups.

### Statistical analysis

As no prior literature exists comparing NVS scores between caregiver groups in a pediatric orthopaedic population, a literature-derived effect size was not available to inform the power calculation. Given that the NVS defines discrete literacy categories at scores of 0-1, 2-3, and 4-6 on a 0-6 scale, a difference that does not shift a caregiver between literacy categories would not be expected to alter clinical management or counseling strategies. The power analysis was therefore designed to detect a large effect size (Cohen's d = 0.80), representing the magnitude of difference most likely to carry clinical significance in this context. A minimum of 25 participants per group was required to detect this clinically-defined differential at 80% power with a two-sided alpha of 0.05.

Bivariate analyses were utilized to compare the cohorts’ baseline caregiver health literacy, PROMIS scores, number of no-shows, number of canceled appointments, and number of advice-only calls. Mann-Whitney U tests were utilized to identify significant differences between the cohorts. Pair-wise post hoc analyses identified specific group differences. A Chi-square test of independence was utilized to examine the association between demographic variables and health literacy levels. Ordinal and logistic regression were used to determine odds ratios of increased NVS scores when controlling for other variables. Multivariate analysis adjusted for known socioeconomic indicators was included. Significance was set at *P* < .05. All analyses were completed using SPSS Statistics (Version 29. IBM Corporation. Armonk, NY, USA). All figures were generated using Prism (Version 10.0. GraphPad Software. San Diego, CA, USA).

## Results

### Demographics

Forty-one percent (37/90) of participants were in the CP group, and 58.89% (53/90) were in the fracture group. The majority of patients were non-White in both groups (CP: 86.1%; fracture: 83.0%, *P* = .694) and of comparable age (CP: mean 10.51 (SD 3.40); fracture: mean 12.13 (SD 4.44), *P* = .065). There were no significant differences when comparing patient sex, caregiver primary language spoken, average caregiver age, caregiver race, insurance type, or ADI between groups (Table 1). Within the CP cohort, Gross Motor Function Classification System (GMFCS) levels ranged from II to V, with the majority of patients classified as GMFCS IV (37.84%) or V (16.22%).

**Table 1:** Descriptive table of patient and caregiver demographics stratified by patient type (N = 90).

Variable∗	CP (n = 37)	Fracture (n = 53)	*P*-value
Caregiver			
*Language*			.806
English	26 (72.2)	37 (69.8)	
Spanish	10 (27.8)	16 (30.2)	
*Race*			.969
White	6 (16.7)	9 (17.0)	
Non-White	30 (83.3)	44 (83.0)	
*Age*	41.34 ± 9.98	39.12 ± 8.77	.281
Patient			
*Biological Sex*			.249
Male	23 (62.2)	39 (73.6)	
Female	14 (37.8)	14 (26.4)	
*Ethnicity*			.129
Hispanic/Latinx	31 (83.8)	37 (69.8)	
Non-Hispanic/Latinx	6 (16.2)	16 (30.2)	
*Race*			.694
White	5 (13.9)	9 (17.0)	
Non-White	31 (86.1)	44 (83.0)	
*BMI*	18.99 ± 5.43	23.69 ± 6.34	<.001
*Age*	10.51 ± 3.40	12.13 ± 4.44	.065
*Insurance*			.377
None	3 (8.1)	1 (1.9)	
Public	30 (81.1)	48(90.6)	
PPO	4 (10.8)	3 (5.7)	
HMO	0 (0.0)	1 (1.9)	
*State ADI*	5.94 ± 1.63	6.00 ± 1.95	.884
*GMFCS Level*			
*II*	13 (35.14%)		
*III*	4 (10.81%)		
*IV* *V*	14 (37.84%) 6 (16.22%)		

∗*Data are presented as frequences, %=valid percentage, or mean±SD. T-test or x*^2^*P-value reported*.

### Baseline health literacy and perceptions of global health

There were no significant differences in caregiver baseline NVS scores between cohorts (CP: mean 3.76 (SD 1.62); fracture: mean 3.55 (SD 2.09), *P* = .790); average scores in both groups corresponded with a “possibility of limited HL.” This trend persisted after ordinal regression was utilized to control for insurance, caregiver race, and caregiver primary language (B Coefficient = 0.100, Standard Error = 0.380, *P* = .792). Baseline PROMIS scores were significantly lower in the CP cohort (CP: mean 41.20 (SD 10.10); fracture: mean 54.58 (SD 8.94), *P* < .001).

### Healthcare utilization

There were several significant differences in healthcare utilization between cohorts (Table 2). The fracture group had significantly higher no-show appointments (CP: mean 0.03 (SD 0.16); fracture: mean 0.49 (SD 1.17), *P* = .008) and more canceled appointments (CP: mean 1.16 (SD 1.69); fracture: mean 1.94 (SD 2.43), *P* = .032) than the CP cohort. Conversely, the CP group had significantly higher advice-only calls (CP: mean 2.51 (SD 3.42); fracture: mean 0.06 (SD 0.31), *P* < .001).

**Table 2:** Bivariate analysis comparing healthcare utilization behaviors between cohorts.

Variable	CP Group (μ, SD)	Fracture Group (μ, SD)	*P*-value∗	Mean Difference∗
Number of No-Shows	0.03 (0.16)	0.49 (1.17)	.008	−.464
Number of advice-only calls	2.51 (3.42)	0.06 (0.31)	<.001	2.457
Number of canceled appointments	1.16 (1.69)	1.94 (2.43)	.032	−.781

∗ *Mann-WhitneyUtest*.

### Socioeconomic and demographic effects on health literacy

For the following analyses evaluating the effects of socioeconomic and demographic factors on health literacy, all subjects were analyzed as a single cohort. There was initially a significant interaction between ADI and HL (*P* = .032). To identify which specific HL groups differed in ADI, post-hoc analysis was conducted which showed no significant difference in ADI between limited literacy and possibility of limited literacy (*P* = .085), possibility of limited literacy and adequate literacy (*P* = .66), or limited literacy and adequate literacy (*P* = 1.00) as demonstrated in Fig. 2.

**Figure 1:**
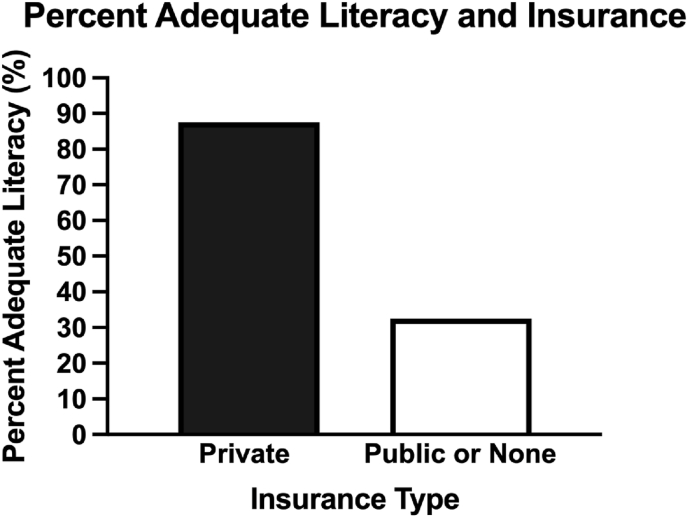
Demonstrates percent of caregivers with adequate literacy (NVS score ≥4) by insurance type (Private: 87.5%, Public or None: 32.5%).

Conversely, bivariate analysis revealed patient ethnicity and insurance status as significant predictors of baseline caregiver HL (Table 3). Caregivers with private health insurance were more likely to have adequate HL (NVS score ≥4) compared to those with public insurance or without insurance (87.5% vs 32.5%). (Fig. 1).

**Table 3:** Bivariate analysis of health literacy by demographic and socioeconomic variables.

	μ (σ)	*P*-value
Sex	Male: 3.42 (1.93) Female: 4.11 (1.79)	.089
Ethnicity	Hispanic: 3.38 (1.81) Non-Hispanic: 4.41 (1.99)	.015
Race	White: 4.27 (2.086) Not White: 3.50 (1.86)	.101
Language	Spanish: 2.95 (1.91) English: 3.84 (1.87)	.067
Insurance	None: 4.75 (1.26) Public: 3.41 (1.89) Private: 5.25 (1.39)	.011

**Figure 2:**
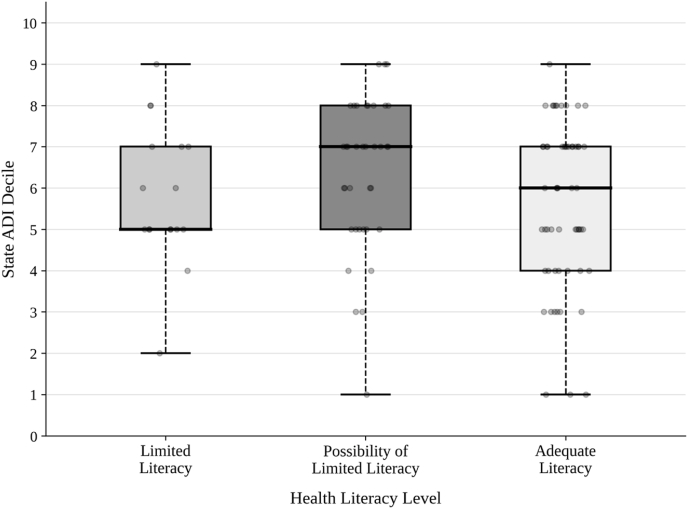
Distribution of State Area Deprivation Index (ADI) by health literacy (HL) group. Boxes represent the interquartile range (Q1–Q3); horizontal line indicates median; whiskers extend to minimum and maximum values within 1.5 × IQR. State ADI decile ranges from 1 (least disadvantaged) to 10 (most disadvantaged). HL categories defined by the Newest Vital Sign (NVS): Limited (score 0–1), Possibility of Limited (score 2–3), Adequate (score ≥4).

Given their significance in bivariate analysis, multivariate analysis including patient ethnicity and insurance status was completed; ADI was additionally included given its role as an established indicator of socioeconomic status. After controlling for patient ethnicity and ADI, the odds ratio for NVS scores for caregivers of patients with private insurance was 6.94 times that of patients with public insurance (log odds = 1.923, SE = 0.874, *P* = .028, Table 4). Adjusting for insurance type and ADI, the odds ratio for NVS scores in caregivers of non-Hispanic patients was 2.74 times that of caregivers for Hispanic patients (log odds = 0.992, SE = 0.480, *P* = .039. Table 3). However, there were no significant differences in caregiver NVS scores in regard to caregiver race (White: mean 4.27 (SD 2.086); Non-White: mean 4.27 (SD 2.086), *P* = .101) or caregiver primary language (Spanish: mean 2.95 (SD 2.086); English: mean 3.84 (SD 1.87), *P* = .067).

**Table 4:** Multivariate regression analysis of NVS scores by demographic and socioeconomic status.

	Odds Ratio	Log Odds	SE	*P*-value
State Area Deprivation Index (1-50 : 51-100)	1.520	0.420	0.115	0.713
Insurance (private: Public)	6.940	1.923	0.874	0.028
Ethnicity (Non-Hispanic/Latinx: Hispanic/Latinx)	2.740	0.992	0.480	0.039

### Sex differences in health literacy

**Figure 3:**
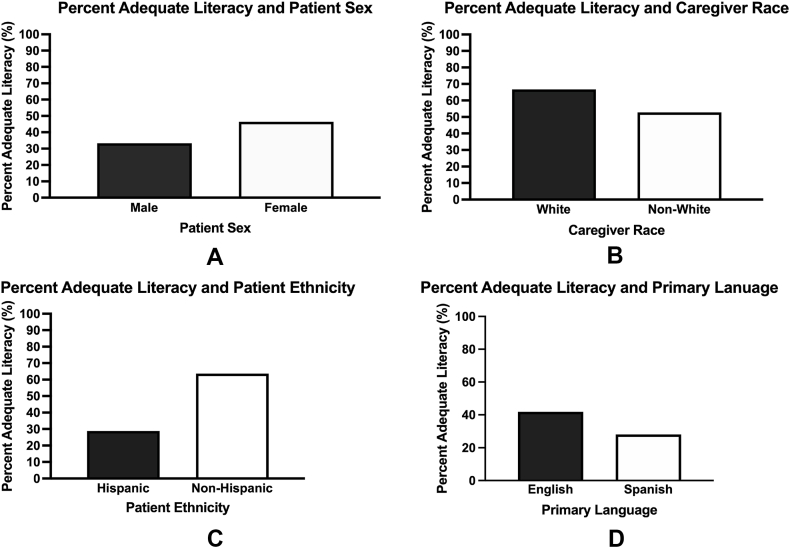
A: Demonstrates the percentage of caregivers with adequate literacy (NVS score ≥4) by patient sex (Male: 33.3%, Female: 46.4%). B: Demonstrates the percentage of caregivers with adequate literacy (NVS score ≥4) by caregiver race (White: 66.7%, Non-White: 52.8%). C: Demonstrates the percentage of caregivers with adequate literacy (NVS score ≥4) by patient ethnicity (Hispanic: 28.8%, Non-Hispanic: 63.6%). D: Demonstrates the percentage of caregivers with adequate literacy (NVS score ≥4) by caregiver primary language (English: 41.9%, Spanish: 28.0%).

When adjusting for insurance status and patient ethnicity, the odds ratio for NVS scores of caregivers of female patients was 2.71 times that of male patients (log odds = 0.998, SE = 0.445, *P* = .025). The percentage of caregivers with adequate health literacy by patient ethnicity, caregiver race, patient sex, and caregiver primary language can be seen in Fig. 3A-3D.

## Discussion

This is the first known study to directly investigate HL of caregivers for children with special healthcare needs [5]. Despite increased healthcare utilization, we found that baseline HL for caregivers of children with a chronic orthopaedic condition is comparable to those with children seeking care for an acute orthopaedic condition. Importantly, mean NVS scores in both cohorts fell within the category of the possibility of limited literacy,' suggesting that neither group achieved adequate literacy. This finding supports a universal precautions approach to health literacy in pediatric orthopaedic care, wherein providers assume a possibility of limited literacy across all caregivers regardless of chronic disease exposure or prior healthcare utilization, rather than attempting to stratify communication strategies based on perceived system familiarity. Moreover, while there was no correlation between ADI, a known indicator of SES, and health literacy, we found specific demographic and socioeconomic indicators of higher HL throughout the entire cohort, including patient female sex, non-Hispanic ethnicity, and private insurance status.

Despite comparable baseline HL levels, caregiver needs, understanding, and utilization of healthcare differed between cohorts. There were higher no-show and cancellation rates in the fracture cohort and greater advice-only calls in the CP cohort, suggesting that HL alone is not a reliable predictor for healthcare utilization. Rather, the severity of disease may be more predictive of healthcare utilization in the pediatric orthopaedic population. Although we did not directly assess disease severity or comorbidity burden, worse perceived global health as reported by caregivers in the CP cohort likely reflects, at least in part, overall disease burden. The significantly lower BMI observed in the CP cohort is consistent with well-established patterns of growth impairment in this population, driven by oral motor dysfunction, dysphagia, and the increased metabolic demands of spasticity, all of which contribute to chronic undernutrition and reduced weight gain in children with CP [[6], [7], [8]]. Increased post-operative calls in the CP cohort likely reflect both the complexity of post-operative care and higher baseline comorbidity burden [5]. This is consistent with prior literature demonstrating that children with CP have higher rates of elective admissions, emergency department visits, and more diagnoses/hospitalizations [[9], [10], [11]]. Our findings further support the need for ongoing education and increased check-ins both pre- and post-operatively to ensure comprehension and caregiver comfort with caring for these complex patients regardless of their familiarity with the medical system.

While our data demonstrated comparable levels of HL among caregivers regardless of healthcare exposure, insurance status–likely a key indicator of socioeconomic status—did correlate with baseline HL. In fact, caregivers of children with private insurance demonstrated significantly higher HL than those with public or no insurance. This association persisted when controlling for other demographic and socioeconomic variables, suggesting that insurance status may reflect HL more accurately than alternative socioeconomic variables. While insurance type is not itself a direct measure of health literacy or a causal determinant of it, it likely functions as a surrogate marker aggregating several underlying determinants including income, educational attainment, and employment stability. More specifically, while ADI is a validated measure of neighborhood-level socioeconomic status, it is derived from aggregate data within a nine-digit postal code. Therefore, while it incorporates multiple socioeconomic factors, it does not directly reflect an individual patient's or family's specific circumstances and should be interpreted as a proxy measure. In contrast, insurance status may more directly represent a patient's socioeconomic position. More expressly, public insurance programs serve populations with lower incomes, limited educational backgrounds, and higher social vulnerabilities, all of which are all of which are independently associated with decreased opportunities for engagement with healthcare providers [12]. In contrast, private insurance is often associated with higher income and educational levels, which provide caregivers with better resources for accessing information and engaging in shared decision making [12]. The observed association between insurance status and health literacy is therefore likely mediated by these upstream socioeconomic factors rather than representing a direct causal relationship. Particularly for individuals in the public insurance system and those without any insurance, resources should be provided to confirm adequate HL, and dedicated time should be allocated to ensure caregiver comprehension prior to and following any surgical procedure.

Caregivers of non-Hispanic patients were three times more likely to have higher NVS scores than those of Hispanic patients. While it may be tempting to attribute the discrepancy in health literacy between caregivers of Hispanic and non-Hispanic patients to a language barrier or caregiver/provider language discordance, primary language (English vs. Spanish) was *not* associated with health literacy. This suggests that the observed differences between Hispanic and non-Hispanic cohorts cannot be explained by language alone. Although all materials were administered in English or Spanish according to caregiver preference, perceptions of bias or stigma may have influenced caregivers' comfort in selecting Spanish materials or completing the assessment in their preferred language. As a result, some caregivers of Hispanic patients may have chosen to complete the NVS in English when they may have been most comfortable with the Spanish version. Moreover, some bilingual Hispanic caregivers may have over-estimated their English proficiency and opted for the English version when they may have been more successful with the Spanish version. Limited familiarity with survey terminology may have contributed to lower scores in this subgroup. In addition, previous research has demonstrated that pediatric healthcare providers incorrectly underestimate the HL of Spanish-speaking caregivers [13,14]. When providers underestimate a caregiver's HL, they might provide overly-simplified explanations, which limits shared decision-making and reduces caregiver engagement. This ultimately hinders opportunities for Spanish-speaking patients and caregivers to improve their HL. This may also subconsciously lead caregivers who primarily speak Spanish to gravitate toward English in healthcare settings, potentially limiting HL and comprehension.

Interestingly, we found that caregivers of female patients demonstrated higher baseline NVS scores compared to caregivers of male patients. While no current studies have directly compared caregiver HL between male and female pediatric patients, previous research has demonstrated significantly higher health care utilization and visit attendance in adolescent females compared to their male counterparts [15,16]. We hypothesize that increased repeated exposure to the healthcare system may lead to higher HL in caregivers of female patients, though this relationship is an exploratory hypothesis and not a definitive conclusion. These caregivers may also become more accustomed to discussing health-related matters, accessing health information, and managing medical care. This finding suggests that, in the absence of major differences in underlying patient complexity, increased interaction with the healthcare system may support incremental gains in caregiver HL. While there were no appreciable differences in HL between the fracture and CP cohorts, this may reflect the confounding influence of substantially greater caregiving complexity in CP, which could attenuate the potential benefits of increased healthcare exposure alone. Our findings therefore suggest that increased healthcare exposure in similarly complex patient populations may, in fact, improve HL. However, this is an exploratory hypothesis. Exposure alone is insufficient to improve HL without considering the broader caregiving context.

This study is not without limitations, including generalizability to the broader pediatric orthopaedic population, as this study was conducted within a single pediatric referral healthcare system and only includes patients treated by fellowship-trained pediatric orthopaedic surgeons in an academic setting. Thus, institutional differences in access to interpreters or educational materials cannot be accounted for. Furthermore, there is potential for selection bias related to recruiting caregivers of only surgical patients and possible response bias when completing health literacy questionnaires in a clinical setting. Additionally, a key limitation of the power analysis performed was that it was designed to detect a clinically meaningful difference in NVS scores corresponding to a shift between literacy categories, rather than a statistically significant marginal difference. As a result, smaller but statistically significant differences in NVS scores between cohorts are not detected with the current analysis. Additionally, while secondary healthcare utilization outcomes reached statistical significance for advice-only calls and no-show appointments, the study may be underpowered for subanalyses given that the power analysis conducted was for the primary outcome only. Caregiver education level, a potential confounder, was also not collected or accounted for. Future studies with larger, stratified cohorts are needed to validate and expand upon these findings as we were only powered for our primary outcome and could not confidently complete further subgroup analysis. Previous analyses found that English speakers were more often categorized as having adequate literacy by NVS compared to the Short Test of Functional Health Literacy in Adults questionnaire while Spanish-speakers scored consistently low on both instruments, suggesting that evaluations of participants’ literacy is likely vary based on HL assessment [17]. Moreover, the PROMIS Global Health Survey was completed by caregivers, who tend to report worse outcomes compared to pediatric patients themselves [18].

## Conclusion

This study emphasizes the need for tailored interventions and educational plans to support caregivers of children with variable health needs and backgrounds and suggests that increased HL should not be implied for caregivers with frequent healthcare system interactions. We suggest that in addition to the standard pre-operative surgical consultation, caregivers of medically-complex patients requiring complex surgical intervention may benefit from augmented educational support and structured follow-up, including interdisciplinary care team meetings, anticipatory guidance, and scheduled phone check-ins, to reduce caregiver anxiety and limit unplanned advice-only calls. Given that mean NVS scores in both cohorts demonstrated the “possibility of limited literacy,” we recommend approaching all caregivers with the assumption that they may have limited literacy or implementing routine screening of pre-operative HL for all pediatric orthopaedic caregivers. Implementing brief validated tools such as the NVS at the time of surgical consultation would allow providers to prospectively identify caregiver literacy levels and tailor the depth, format, and complexity of pre- and post-operative education accordingly, facilitating a more personalized approach to caregiver communication. Our findings emphasize the importance of providing education and resources at each visit, ensuring caregiver comprehension regardless of patient status, system familiarity or healthcare utilization rates.

## Author contributions

**Nora A. Galoustian:** Writing – review & editing, Writing – original draft, Project administration, Methodology, Investigation, Formal analysis, Data curation, Conceptualization. **Michaela Juels:** Writing – original draft, Project administration, Methodology, Investigation, Data curation, Conceptualization. **Aura Elias:** Writing – review & editing, Data curation. **Gabrielle Noullet:** Writing – review & editing, Investigation, Data curation. **Anahit Malumyan:** Writing – original draft, Methodology, Investigation, Data curation. **Lisa Su:** Writing – review & editing, Writing – original draft, Investigation, Conceptualization. **Rachel Mednick Thompson:** Writing – review & editing, Writing – original draft, Project administration, Methodology, Investigation, Data curation, Conceptualization.

## Ethics approval and consent

The author(s) declare that no patient consent was necessary as no images or identifying information are included in the article.

## Funding

There are no conflicts of interests or sources of funding to disclose.

## Declaration of competing interests

The authors declare the following financial interests/personal relationships which may be considered as potential competing interests: Rachel Mednick Thompson reports a relationship with Arthrex Inc that includes: consulting or advisory. If there are other authors, they declare that they have no known competing financial interests or personal relationships that could have appeared to influence the work reported in this article.
